# Post-error Slowing Reflects the Joint Impact of Adaptive and Maladaptive Processes During Decision Making

**DOI:** 10.3389/fnhum.2022.864590

**Published:** 2022-06-09

**Authors:** Fanny Fievez, Gerard Derosiere, Frederick Verbruggen, Julie Duque

**Affiliations:** ^1^Institute of Neuroscience, Université catholique de Louvain, Brussels, Belgium; ^2^Department of Experimental Psychology, Ghent University, Gent, Belgium

**Keywords:** speed-accuracy tradeoff, error processing, cognitive control, attention, emotion

## Abstract

Errors and their consequences are typically studied by investigating changes in decision speed and accuracy in trials that follow an error, commonly referred to as “post-error adjustments”. Many studies have reported that subjects slow down following an error, a phenomenon called “post-error slowing” (PES). However, the functional significance of PES is still a matter of debate as it is not always adaptive. That is, it is not always associated with a gain in performance and can even occur with a decline in accuracy. Here, we hypothesized that the nature of PES is influenced by one’s speed-accuracy tradeoff policy, which determines the overall level of choice accuracy in the task at hand. To test this hypothesis, we had subjects performing a task in two distinct contexts (separate days), which either promoted speed (hasty context) or cautiousness (cautious context), allowing us to consider post-error adjustments according to whether subjects performed choices with a low or high accuracy level, respectively. Accordingly, our data indicate that post-error adjustments varied according to the context in which subjects performed the task, with PES being solely significant in the hasty context (low accuracy). In addition, we only observed a gain in performance after errors in a specific trial type, suggesting that post-error adjustments depend on a complex combination of processes that affect the speed of ensuing actions as well as the degree to which such PES comes with a gain in performance.

## Introduction

We all make mistakes. For instance, many of us have experienced sending an email to the wrong person. After such an error, we typically write a second message to apologize and rectify. But when we send the second email, we usually take more time to check that the recipient is correct. We, therefore, adapt our behavior in order to avoid reproducing previous mistakes. Such an ability to adapt after an error is essential to achieving our goals.

Errors and their consequences are typically studied in two-choice reaction time tasks by investigating changes in decision speed and accuracy in trials that follow an error, commonly referred to as “post-error adjustments”. Using such tasks, many studies have reported that subjects slow down following an error, a phenomenon called “post-error slowing” (PES; Fu et al., 2019; Dubravac et al., 2020; Nigbur and Ullsperger, 2020; Topor et al., 2021).

The functional significance of PES is still a matter of debate though (Wessel, 2018; Damaso et al., 2020; Kirschner et al., 2021). Because slowing down after an error is often associated with an increase in accuracy, PES is traditionally attributed to adaptive adjustments of decision policies, favoring a more cautious response style to improve performance in the subsequent trial (Rabbitt and Vyas, 1970; Smith and Brewer, 1995; Cavanagh et al., 2014; Siegert et al., 2014; Purcell and Kiani, 2016; Steinhauser and Andersen, 2019; Beatty et al., 2020). However, several recent studies have revealed that PES can also occur in a somewhat “maladaptive” way as, sometimes, slowing does not necessarily lead to improvement in accuracy; in fact, PES can even come with a decrease in decision accuracy (Ceccarini et al., 2019; Eben et al., 2020a; Schroder et al., 2020; Smith et al., 2020; Compton et al., 2021; Kirschner et al., 2021). These findings indicate that the functional significance of PES may vary according to the context in which it is observed.

A careful analysis of the literature reveals that the degree to which PES is adaptive (i.e., increases accuracy) or “maladaptive” (i.e., takes place without accuracy improvement) depends partly on the average level of accuracy of subjects in the task at play. That is, in studies reporting an adaptive PES, the overall level of choice accuracy is typically low (i.e., generally between 60% and 80% of correct choices) because the task is relatively complex and/or because the instruction requires subjects to respond quickly within a given time limit (Siegert et al., 2014; Purcell and Kiani, 2016; Steinhauser and Andersen, 2019). In this situation, errors are clearly expected and slowing down after them has a positive effect on choice accuracy (Hajcak et al., 2003; Siegert et al., 2014; Dyson et al., 2018; Wessel, 2018; Damaso et al., 2020). By contrast, studies reporting a maladaptive PES rather use reaction time tasks that are quite simple such that the overall level of choice accuracy is usually much higher (i.e., more between 80% and 100% of correct choices; Notebaert et al., 2009; Nunez Castellar et al., 2010; Houtman et al., 2012; Eben et al., 2020a; Li et al., 2020; Kirschner et al., 2021; Compton et al., 2021). In such settings, errors represent infrequent and unexpected events that may catch attention, resulting in a maladaptive PES that deteriorates (rather than enhances) choice accuracy in the consecutive trial (Sokolov, 1963; Nunez Castellar et al., 2010; Houtman et al., 2012).

Thus, whether PES is adaptive or maladaptive might be partly influenced by choice accuracy. This in turn depends on the task characteristics, such as its global difficulty or on the speed-accuracy tradeoff (SAT) policy of subjects performing the task. Indeed, most decisions require balancing speed and accuracy, making the SAT a universal property of behavior (Henmon, 1911; Rinberg et al., 2006; Salinas et al., 2014; Guo et al., 2020; Reynaud et al., 2020; Miletić et al., 2021). Humans and other non-human animals are able to adjust their SAT depending on the context, favoring either hasty (i.e., high speed, low accuracy) or cautious (i.e., low speed, high accuracy) decision policies (Chittka et al., 2009; Heitz, 2014; Spieser et al., 2017; Thura, 2020). Hence, because choice accuracy varies depending on the SAT, it is plausible that PES can shift from being adaptive to being maladaptive depending on whether the emphasis is on speed or accuracy when performing the same task in separate blocks.

In conclusion, past research suggests that errors can trigger PES of adaptive or maladaptive nature (van Driel et al., 2012; Schiffler et al., 2017; Wessel, 2018). These two different types of behavior have been evidenced in separate studies using distinct tasks or instructions where performance is either characterized by a low or a high level of choice accuracy, respectively. Here, we hypothesized that the nature of PES can also vary within a given task depending on whether the SAT context favors a hasty (i.e., high speed, low accuracy) or a cautious (i.e., low speed, high accuracy) decision policy. More precisely, we predicted that errors would be common and expected when the context favors choice speed due to the choices’ promptness (Damaso et al., 2020), whereas they would be rare and unexpected when the context favors choice accuracy. Hence, we expected PES to be less adaptive (and potentially maladaptive) when the emphasis is on choice accuracy in a cautious SAT context compared to when the emphasis is on response speed. To test this hypothesis, we used a modified version of the “tokens task” (Cisek et al., 2009; Derosiere et al., 2019, 2022), involving choices between left and right index fingers. In this task, incorrect choices led either to a *low* or *high* penalty in two different SAT contexts, inciting subjects to implement either *hasty* or *cautious* decision policies, respectively. We predicted that PES would be more adaptive (i.e., associated with a higher increase in accuracy) in the low than in the high penalty context.

## Material and Method

### Participants

A total of 43 healthy volunteers participated in this study (25 Women: 23.5 ± 2.3 years old). All participants were right-handed according to the Edinburgh Questionnaire (Oldfield, 1971). None of them had any neurological disorder or history of psychiatric illness or drug or alcohol abuse, and no one was following any clinical treatment that could have influenced performance. Participants were financially compensated for their participation and could also receive extra compensation based on their performance on the task (see below). All gave written informed consent at the beginning of the experiment. The protocol was approved by the Ethics Committee of the Université catholique de Louvain (UCLouvain), Brussels, Belgium. Data presented here were also used (for a different purpose) in another article (Derosiere et al., 2022).

### Tokens Task

**Figure 1:**
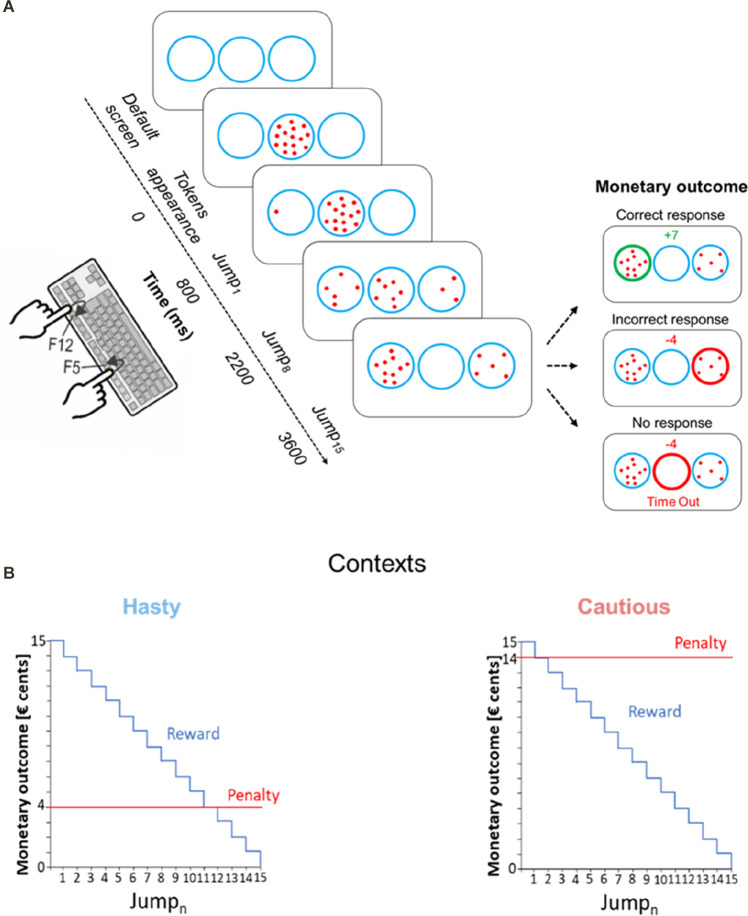
**(A)** Schematic of the tokens task. In each trial, 15 tokens jumped one by one every 200 ms from the central circle to one of the lateral circles. The subjects had to indicate by a left or right index finger keypress (i.e., F12 and F5 keys, respectively) which lateral circle they thought would receive the majority of tokens at the end of the trial. For a correct response, the subjects won, in € cents, the number of tokens remaining in the central circle at the time of the response. Hence, the reward earned for a correct response decreased over time, as depicted in **(B)**. The right side of panel **(A)** depicts the monetary outcome in three exemplary cases. The upper inset represents the reward provided for a correct response between Jump_8_ and Jump_9_, that is when seven tokens remain in the central circle at the moment the left circle is chosen; the middle inset represents the penalty for an incorrect response in the hasty context, fixed at −4 cents; the lower inset shows the penalty in a “Time Out” trial (no response), fixed at −4 cents, regardless of the context. For representative purposes, the “Time Out” message is depicted below the circles in this example, while it was presented on top of the screen in the actual experiment. **(B)** Contexts. Incorrect responses led to a fixed negative score, which differed depending on the context. In the hasty context (shown on the left), the penalty was low, equaling only 4 cents (see red line), promoting fast decisions. In contrast, in the cautious context (shown on the right), the penalty was high, equaling 14 cents, promoting thus slower decisions.

Subjects were seated in front of a computer screen, positioned at a distance of 70 cm from their eyes. Both forearms were placed on the surface of a table with the left and right index fingers placed on a keyboard turned upside down (Figure 1A). Subjects performed a variant of the “tokens task”(Cisek et al., 2009; Thura and Cisek, 2014; Derosiere et al., 2021) which was implemented by means of LabView 8.2 (National Instruments, Austin, TX). In this decision-making task, participants had to continuously monitor the distribution of 15 tokens jumping one by one from a central circle to one of two lateral circles. The subjects were instructed to guess which lateral circle would ultimately receive the majority of the tokens; they had to indicate their choice before the last token jump, by pressing a key with the left or right index finger (i.e., an F12 or F5 key-press for the left or right circle, respectively).

As depicted in Figure 1A, in between trials, subjects were always presented with a default screen, consisting of three blue circles (4.5 cm diameter each) displayed on a white background for 2,500 ms. Each trial started with the appearance of the 15 tokens randomly arranged in the central circle. After a delay of 800 ms, a first token jumped towards the left or right circle, followed every 200 ms, by the other tokens, jumping one by one, to one of the two lateral circles. Subjects were asked to respond as soon as they felt sufficiently confident. The reaction time (RT) was calculated by computing the difference between the time at which subjects pressed the key to indicate their choice and the time of the first tokens jump (Jump_1_). After subjects had pressed the corresponding key, the tokens kept jumping every 200 ms until the central circle was empty (i.e., 2,800 ms after Jump_1_). So, the feedback appeared only once all tokens were distributed. At this time, the chosen circle was highlighted either in green or in red depending on whether the response was correct or not, respectively. In addition, a numerical score displayed above the central circle provided subjects with feedback of their performance (see the “Reward, Penalty and SAT contexts” section below). In the absence of any response before the last jump, the central circle turned red with a “Time Out” message and a “−4” (score) appeared on top of the screen. The feedback screen lasted for 500 ms and then disappeared at the same time as the tokens did (the circles always remained on the screen), denoting the end of the trial. From the appearance of the tokens in the central circle, each trial lasted for 6,600 ms.

One key feature of the tokens task is that it allows one to calculate, in each trial, the “success probability” *p_i_ (t)* associated with choosing the correct circle *i* at each moment in time *t*. For example, for a total of 15 tokens, if at a particular moment in time the right *(R)* circle contains *NR* tokens, the left *(L)* circle contains *NL* tokens, and the central *(C)* circle contains *NC* tokens, then the probability that the circle on the left will ultimately be the correct one (i.e., the success probability of guessing left) is described as follows, where k represents the number of elements in the summation component of the equation:


(1) $$p\;\left(L|NL,NR,NC\right)=\frac{NC!}{2^{NC}}\sum_{k\;=\;0}^{\min(NC,7-NR)}\frac{1}{k!\;\left(NC-k\right)!}$$

Although the token jumps appeared completely random to subjects, the direction of each jump was determined a priori, producing different types of trials according to specific temporal profiles of pi(t). There were four trial types: ambiguous, obvious, misleading, and arbitrary. The majority of trials (60%) were ambiguous, as the initial jumps were balanced between the lateral circles, keeping the p_i_(t) close to 0.5 until late in the trial (i.e., p_i_(t) remained between 0.5 and 0.66 up to the Jump_10_). Fifteen percentage of trials were “obvious”, meaning that the initial token jumps consistently favored the correct circle (i.e., p_i_(t) was already above 0.7 after Jump_3_ and above 0.8 after jump_5_). Fifteen percentage of the trials were “misleading”, where most of the first token jumps occurred towards the incorrect lateral circle (i.e., p_i_(t) remained systematically below 0.4 until Jump_3_; from then on, the following tokens jumped mainly in the other direction, that is, towards the circle that eventually turned out being correct). Finally, we included 10% of trials that were completely arbitrary. These different types of trials were always presented in a randomized order.

### Reward, Penalty, and SAT Contexts

As mentioned above, at the end of each trial, subjects received a feedback score. Correct responses led to a positive score (i.e., a reward) while incorrect responses led to a negative score (i.e., a penalty). Subjects were told that the sum of these scores would turn into a monetary reward at the end of the experiment.

In correct trials, the reward corresponded to the number of tokens remaining in the central circle at the time of the response (in € cents). Hence, the reward for a correct choice in a given trial gradually decreased over time (Figure 1B). For instance, a correct response provided between Jump_5_ and Jump_6_ led to a gain of 10 cents (10 tokens remaining in the central circle). However, it only led to a gain of 5 cents when the response was provided between Jump_10_ and Jump_11_ (5 tokens remaining in the central circle). Hence, using a reward dropping over time increased time pressure over the course of a trial and pushed subjects to respond as fast as possible (Derosiere et al., 2019, 2022).

The penalty provided for incorrect choices did not depend on the time taken to choose a lateral circle. Importantly though, it differed between the two contexts. In the first context, the cost of making an incorrect choice was low as the penalty was only −4 cents, pushing subjects to make hasty decisions in order to get high reward scores (hasty context). Conversely, incorrect choices were severely sanctioned in the second context as the penalty there was −14 cents, emphasizing the need for cautiousness (cautious context).

Moreover, not providing a response before Jump_15_ (i.e., time out trials) also led to a penalty, which was −4 cents both in the hasty and in the cautious contexts. Hence, in the hasty context, providing an incorrect response or not responding led to the same penalty (i.e., −4 cents), further increasing the urge to respond before the end of the trial in this context. Conversely, in the cautious context, the penalty for making an incorrect choice was much higher than that obtained for an absence of response (i.e., −14 vs. −4 cents, respectively), further increasing subjects’ cautiousness in this context.

Hence, with these two contexts, we could consider post-error behavioral adjustments depending on whether the cost of errors was either low or high, prompting the subjects to put the emphasis on decision speed (low accuracy) or on decision accuracy (high accuracy), respectively. As mentioned above, we expected to observe a post-error slowing (PES) in both cases but predicted that it would be more adaptive in the hasty than in the cautious blocks.

### Sensory Evidence at RT

The tokens task also allowed us to assess the amount of sensory evidence (i.e., available information) supporting the subjects’ choice at the RT. To estimate the level of sensory evidence at RT, we computed a first-order estimation as the sum of log-likelihood ratios (SumLogLR) of individual token movements at this time (Cisek et al., 2009):


(2) $$SumLogLR\;(n)=\sum_{k\;=\;1}^{n}\log\frac{p\;\;\left(ek|S\right)}{p\;\left(ek|NS\right)}$$

In this equation, p(e_k_|S) is the likelihood of a token event e_k_ (a token jumping into either the chosen or unchosen lateral circle) during trials in which the chosen circle S is correct, and p(e_k_|NS) is its likelihood during trials in which the unchosen circle NS is correct. K, here, represents the different token jumps. The SumLogLR is proportional to the difference between the number of tokens contained in each lateral circle; the larger the number of tokens in the chosen circle, as compared to the unchosen circle, the higher is the evidence for the choice and thus the SumLogLR (Derosiere et al., 2019). We expected the latter to be overall higher in the cautious than in the hasty context, reflecting the higher evidence needed before committing to an accurate choice in the former context (Ratcliff, 2002; Heitz, 2014; Miletić et al., 2021).

### Experimental Procedure

Subjects performed the task in the two contexts in two different experimental sessions conducted on separate days at a 24-h interval. The order of the two sessions (i.e., hasty and cautious) was counterbalanced across participants. As described below, each session involved the same structure, except for the addition of a familiarization block in the first session only, to allow subjects to become acquainted with the basic principles of the task (this was of course not necessary for the session coming on the 2nd day).

Each session started with two short blocks involving a simple reaction time (SRT) task. This task was similar to the tokens task described above except that, here, all tokens jumped simultaneously into one of the two lateral circles. The subjects were instructed to respond as fast as possible by pressing the appropriate key (i.e., F12 or F5 for the left or the right circle, respectively). In a given SRT block, the tokens jumped always into the same circle, and subjects were informed in advance of the circle to choose within a block. This SRT task allowed us to estimate the sum of the delays attributable to the sensory and motor processes in the absence of a choice, as achieved in past studies (Cisek et al., 2009; Thura et al., 2014).

Then, subjects performed a few practice blocks. The first one (10 trials) consisted of a version of the tokens task in which the feedback was simplified; indicating only if the subjects’ choice was correct or incorrect by highlighting the chosen circle in green or red, respectively; no reward or penalty was provided here. This first practice block served to familiarize subjects with the basic aspects of the task and was only used during the first session. The practice then continued with two blocks (20 trials each) where subjects performed the task in the context they would be involved in for the whole session (hasty or cautious blocks).

After that, the actual experiment involved eight blocks of 40 trials (320 trials per session; 640 trials per subject). Each block lasted about 4 min and a break of 2–5 min was provided between each block. Each session lasted approximately 150 min.

### Statistical Analyses

The analyses comprised two parts: first, we ran some tests to check that our manipulation of the penalty indeed led the subject to adopt different SAT policies in the two contexts. Second, and more related to the goal of the current study, we performed analyses to compare the post-error adjustments in the two contexts. Most of the statistical comparisons involved repeated-measures analyses of variance (ANOVA_RM_) run with the Statistica software (version 10.0, Statsoft, Oklahoma United-States). *Post hoc* comparisons were conducted using the Tukey’s Honestly Significant Difference (HSD) procedure. The significance level was set at *p* < 0.05. Moreover, for the analyses regarding post-error adjustments, we ran a Bayesian equivalent of the ANOVA_RM_ (and *t*-tests) with JASP (Wagenmakers et al., 2018). In this case, the Bayes Factor (BF_10_) quantifies the evidence for the alternative hypothesis against the null hypothesis and the prior and posterior inclusion probabilities [P(incl) et P(incl|data)] refer to the importance of each parameter based on the prior and posterior probabilities of each model including it, respectively. All data are presented as mean ± SE.

#### Manipulation Check

In order to verify that our manipulation of penalty (−4 or −14 cents) successfully induced SAT adaptations, we considered the RT, the percentage of correct choices (%Correct), and the SumLogLR at RT in the two contexts. Overall, we expected to observe larger values for these variables in the high penalty (−14 cents) than in the low penalty (−4 cents) blocks, supporting a more conservative behavior in the cautious context compared to the hasty one. To address this directly, we analyzed each variable using two-way ANOVAs_RM_ with CONTEXT (hasty or cautious) and TRIAL_TYPE (obvious, ambiguous, or misleading) as within-subject factors.

#### Post-error Adjustments

All analyses on post-error adjustments focused on behavior in ambiguous trials. This allowed us to characterize post-error adjustments in a homogeneous set of (ambiguous) trials. We investigated behavior in these trials, referred to as the “n” trials (trials_n_), according to whether they followed an error or a correct choice. These trials preceding trials_n_ are referred to as trial_n-1_ and were separated according to whether they were ambiguous or misleading; there were too few errors in obvious trials to consider them as trials_n-1_. Thus, we considered post-error adjustments on ambiguous trials_n_ according to the type of trials_n-1_ (ambiguous or misleading). For this analysis, we had to exclude 14 participants who had less than five trials_n_ in at least one of the experimental conditions. As a result, statistical analyses were run on a total of 29 subjects (17 women: 23.4 ± 2.4 years old). On average in the hasty context, we characterized adjustments following errors in 22 ± 8 ambiguous trial_n-1_ and 15 ± 6 misleading trial_n-1_ (corresponding to an error rate of 21 ± 7% and 44 ± 18%, respectively). In the cautious context, errors occurred in 13 ± 5 ambiguous trials_n-1_ and 10 ± 4 misleading trial_n-1_ (corresponding to an error rate of 13 ± 5% and 30 ± 12%, respectively).

There are different methods for quantifying post-error adjustments in trials_n_ (Hajcak and Simons, 2002; Dutilh et al., 2012a). In the present study, we used a traditional approach consisting in calculating deltas (Δ) for the RT (ΔRT, ms) and for the % Correct (Δ%Correct) in trials_n_ as follows: ΔRT was obtained by calculating the difference between the RT in correct trials_n_ that either followed an error or a correct choice in trials_n-1_ (Williams et al., 2016; Damaso et al., 2020; Smith et al., 2020). Similarly, Δ%Correct corresponded to the difference in % Correct between trials_n_ following an error or a correct choice in trials_n-1_. Hence, PES manifests as a positive ΔRT. If this positive ΔRT is associated with a positive Δ%Correct, it means that the PES is adaptive (i.e., is associated with a gain in decision accuracy) while a null or negative Δ%Correct reflects a maladaptive PES (no gain or drop in decision accuracy). These ΔRT and Δ%Correct were analyzed using two-ways ANOVAs_RM_ with CONTEXT (hasty or cautious) and trials_n-1_-TYPE (ambiguous or misleading) as within-subject factors.

## Results

### Manipulation Check

**Figure 2:**
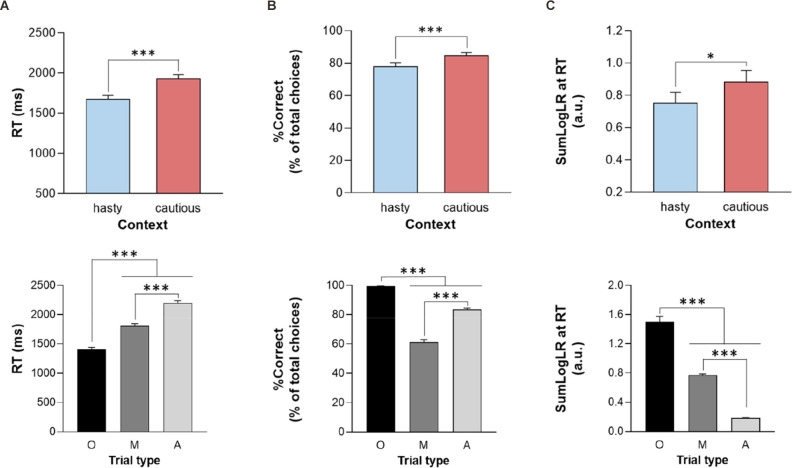
Reaction time (**A**; RT), percentage of correct choices (**B**; %Correct), and sensory evidence at RT (**C**; SumLogLR at RT), depending on the context (upper panel; hasty or cautious), and the trial type [lower panel; Obvious (O), Misleading (M) or Ambiguous (A)]. Error bars represent SE. ^*^p < 0.05, ^***^p < 0.001: significantly different.

On average, subjects displayed RTs of 1866 ± 457 ms; they performed with a %Correct of 81 ± 18%, and did so for a level of evidence corresponding to 0.35 ± 0.72 (SumLogLR at RT value; a.u.). Importantly, as depicted in Figure 2 (upper panel), all these values were lower when the penalty was low (i.e., equal to −4 cents) compared to when it was high (i.e., equal to −14 cents), supporting a shift from a cautious to a hasty response policy when the penalty was low (all CONTEXT *F*_1, 28_ > 7.3, all *p* < 0.05, see Table 1).

**Table 1:** Inferential analyses of behavioral adaptations to the SAT context.

	Factor	MES	*F*-value	*p*-value	*η*_p_^2^
**RT**	CONTEXT	2,903,696	33.667	<0.001	0.987
	TRIAL_TYPE	9,316,608	385.188	<0.001	0.546
	CONTEXT X TRIAL_TYPE	6,143	0.582	<0.562	0.932
**%Correct**	CONTEXT	2,117	41.31	<0.001	0.999
	TRIAL_TYPE	21,663	222.91	<0.001	1
	CONTEXT X TRIAL_TYPE	655	14.35	<0.001	0.998
**SumLogLR at RT**	CONTEXT	0.745	7.349	0.011	0.208
	TRIAL_TYPE	25.172	222.121	<0.001	0.888
	CONTEXT X TRIAL_TYPE	0.260	2.728	0.074	0.089

*The main error square (MES), critical F-value, p-value, and partial eta-squared (*η*_p_^2^) are provided for each factor (CONTEXT; TRIAL_TYPE) and their interaction (CONTEXT × TRIAL_TYPE), following the analysis of the reaction time (RT), percentage of correct choices (%Correct), and sensory evidence at RT (SumLogLR at RT). Significant p-values are highlighted in bold and blue*.

In addition, as shown on the lower panel of Figure 2, the ANOVA_RM_ revealed an effect of the TRIAL_TYPE on all three parameters (all *F*_2, 56_ > 222, *p* < 0.001). As expected, subjects responded faster and more accurately in the obvious trials than in the other trials (all *p* < 0.001). They were also faster in misleading than in ambiguous trials (*p* < 0.001) but showed a lower accuracy (i.e., lower %Correct) in the former trial type (*p* < 0.001), consistent with their misleading nature. Regarding the SumLogLR at RT, it was the highest in the obvious and the lowest in the ambiguous trials (all *p* < 0.001), consistent with the different predefined patterns of token jumps in these different trial types.

**Figure 3:**
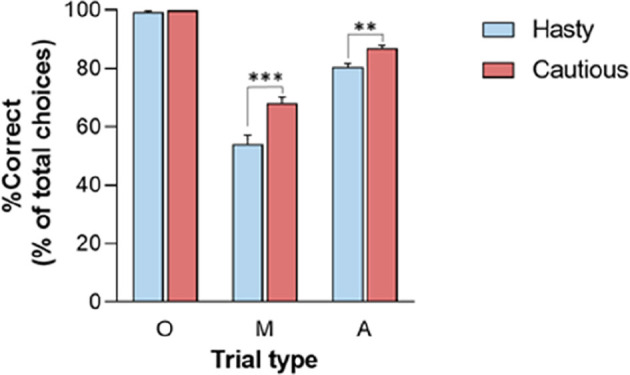
CONTEXT × TRIAL_TYPE interaction on the percentage of correct choices (%Correct).%Correct was lower in the hasty (blue bars) than in the cautious (red bars) context when considering Misleading (M) and Ambiguous (A) trials but not for the Obvious (O) trials. Note the absence of errors in these latter trials (%Correct = 100), whether in the hasty or cautious context. ^**^p < 0.01, ^***^p < 0.001: significantly different.

Finally, the RT and SumLogLR at RT did not display any significant CONTEXT × TRIAL_TYPE interaction (all *F*_2, 56_ < 2.8, all *p* > 0.05). Yet, as depicted in Figure 3, this interaction was significant for the %Correct (*F*_2, 56_ = 14.35, *p* < 0.001). As such, the %Correct was larger in the cautious context relative to the hasty one but only in ambiguous and misleading trials (*p* < 0.01 and *p* < 0.001, respectively). In fact, the obvious trials were so easy that subjects did not make mistakes in this trial type whatever the context.

### Post-error Adjustments

**Figure 4:**
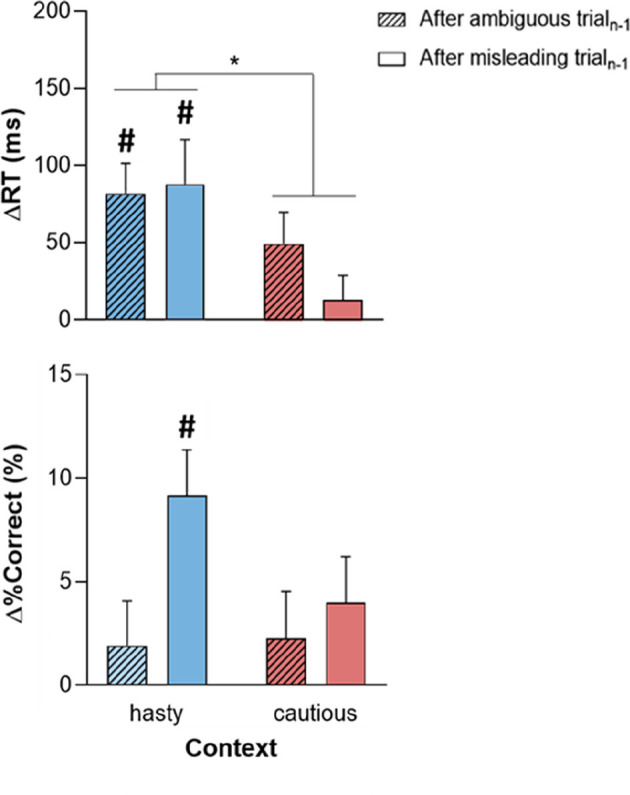
Post-error adjustments of reaction time (ΔRT; upper panel) and %Correct (Δ%Correct; lower panel) depending on the context (hasty or cautious) and on whether trial_n-1_ was ambiguous (crosshatched bars) or misleading (empty bars). While the positive ΔRT in all conditions suggests the presence of PES, this slowing down was only significant in the hasty context. The latter PES came with a positive Δ%Correct but this effect was only significant following misleading trials_n-1_. Error bars represent SE. ^#^: *t*-test against 0 (significant difference from 0). *p < 0.05: significantly different.

Post-error adjustments (ΔRT and Δ%Correct), calculated with the traditional approach (Dutilh et al., 2012a), are displayed in Figure 4 for trials_n_ (always ambiguous), following either ambiguous or misleading trials_n-1_. Even if ΔRTvalues were positive in all conditions, which would be consistent with the occurrence of PES, Student’s *t*-tests against 0 showed that this slowdown was only significant in the hasty context (ΔRT significantly above 0 with a Bonferroni-corrected threshold of 0.05/4), regardless of the TRIAL_n-1__TYPE (both *t*_29_ > 3, *p* = [0.0003 0.005], Cohen’s d = [0.567 0.767]); *t*-tests did not reveal any significant difference between ΔRT and 0 in the cautious context (both TRIAL_n-1__TYPE *t*_29_ > 0.8, *p* = [0.024 0.401], Cohen’s d = [0.158 0.444]). Similarly, equivalent Bayesian analyses (Wagenmakers et al., 2018) showed moderate to strong evidence for PES in the hasty context (BF_10_ = [8.330 98.677]), and moderate evidence for a lack of adjustment after an error in the caution context (BF_10_ = [0.275 2.207]). Consistently, the ANOVA_RM_ revealed a significant effect of CONTEXT on ΔRT (*F*_1, 28_ = 6.26, *p* = 0.018), in the absence of TRIAL_n-1__TYPE effect (*F*_1, 28_ = 0.49, *p* = 0.49) or CONTEXT × TRIAL_n-1__TYPE interaction (*F*_1, 28_ = 1.39, *p* = 0.25). These results were supported by a Bayesian analysis showing moderate evidence for a context effect (BF_10_ = 5.411, see Table 2).

**Table 2:** Inferential analyses of behavioral changes in trial_n_.

	**Factor**	**P(incl)**	**P(incl|data)**	**BF**_incl_	**BF**_10_	*η*_p_^2^
Δ**RT**	CONTEXT	0.600	0.853	3.854	5.411	0.183
	TRIAL_n-1__TYPE	0.600	0.247	0.219	0.255	0.17
	CONTEXT X TRIAL_n-1__TYPE	0.200	0.065	0.278	0.553	0.05
Δ**%Correct**	CONTEXT	0.600	0.322	0.317	0.348	0.051
	TRIAL_n-1__TYPE	0.600	0.626	1.116	1.444	0.112
	CONTEXT X TRIAL_n-1__TYPE	0.200	0.078	0.341	0.283	0.050

*The prior inclusion probability P(incl), the posterior inclusion probability P(incl|data), and the change from prior to posterior inclusion odds (BF_incl_) are provided for each factor (CONTEXT; TRIAL_n-1__TYPE) and their interaction (CONTEXT × TRIAL_n-1__TYPE), following the analysis of reaction time and %Correct change in trial_n_ (ΔRT and Δ%Correct). In addition, the BF_10_ grades the strength of evidence for the alternative hypothesis against the null hypothesis and the partial eta-squared (*η*_p_^2^) represents a measure of the effect size. BF_10_ revealing a significant factor effect is highlighted in bold and blue*.

Figure 4 (lower panel) evokes a positive Δ%Correct in all conditions, which would indicate an increase in decision accuracy in trial_n_. Yet, the Student’s *t*-tests showed that this effect was only significant in the hasty context; more surprisingly, it was only present following misleading trials_n-1_ (*t*_29_ = 4.21, *p* < 0.001, Cohen’s d = 0.781 and BF_10_ = 118.923; Bonferroni-corrected threshold = 0.05/4, see Table 3 for more details). Note though that the variations in Δ%Correct between the different conditions were rather weak, as confirmed by the ANOVA_RM_ analyses which only revealed a marginal effect of TRIAL_n-1__TYPE (*F*_1, 28_ = 3.53, *p* = 0.07), with no effect of CONTEXT (*F*_1, 28_ = 1.51, *p* = 0.23) or CONTEXT × TRIAL_n-1__TYPE interaction (*F*_1, 28_ = 1.48, *p* = 0.23 and BF_10_ = 0.283). These results were supported by a Bayesian analysis showing anecdotal evidence for a TRIAL_n-1__TYPE effect (BF_10_ = 1.444, see Table 2).

**Table 3:** Inferential Student’s *t*-tests of behavioral changes in trial_n_.

	**Factor**	*t*-value	***p*-value**	**Cohen’s d**	BF_10_
Δ**RT**	after ambiguous trials_n-1_ in hasty context	4.131	<0.001	0.767	98.677
	after misleading trials_n-1_ in hasty context	3.053	0.005	0.567	8.330
	after ambiguous trials_n-1_ in cautious context	2.390	0.024	0.444	2.207
	after misleading trials_n-1_ in cautious context	0.853	0.401	0.158	0.275
Δ**%Correct**	after ambiguous trials_n-1_ in hasty context	0.894	0.379	0.166	0.284
	after misleading trials_n-1_ in hasty context	4.208	<0.001	0.781	118.923
	after ambiguous trials_n-1_ in cautious context	0.994	0.329	0.185	0.310
	after misleading trials_n-1_ in cautious context	1.772	0.087	0.329	0.786

*The critical t-value, the p-value, and the Cohen’s d as a measure of the effect size are represented for each factor (after ambiguous or misleading trials_n-1_ in hasty or cautious context), following the analysis of reaction time and %Correct change in trial_n_ (ΔRT and Δ%Correct). Significant p-value (with a Bonferroni-corrected threshold of 0.05/4) are highlighted in bold and blue*.

In conclusion, our data indicate that post-error adjustments varied according to the context in which subjects performed the tokens task, with PES being only significant in the hasty context, and a gain in performance being only observed after errors in misleading trials.

## Discussion

The literature on post-error adjustments is quite diverse and controversial, especially regarding the nature of PES; although often adaptive, PES sometimes comes with a decline in accuracy, suggesting that it can be maladaptive in some instances. Here, we investigated if the nature of PES can vary according to whether a subject behaves in a context favoring hasty or cautious decisions. To address this point, we had subjects perform the tokens task in separate blocks where errors were either poorly penalized, encouraging hasty responses (but low accuracy), or highly penalized, calling for more cautiousness (at the cost of speed). The results show that, overall, subjects slowed down after erroneous choices, supporting the presence of PES. Yet, despite the fact that ΔRT values were numerically positive in all conditions, this PES was only significant in the hasty context (after correction for multiple comparisons). Moreover, consistent with an adaptive adjustment in this context, we observed a significant improvement in performance, but only following misleading trials_n-1_; the positive Δ%Correct did not reach significance following ambiguous trials_n-1_.

The positive values of ΔRT in all conditions indicate that, if anything, subjects slowed down after an error. However, contrary to our expectation to observe PES in the two contexts, this ΔRT was only significant in the hasty context suggesting that subjects only slowed down when they were in a context emphasizing speed (low accuracy) but not when the context promoted more accurate choices. PES, as observed in the hasty context, is usually associated with a cognitive control process recruited to prevent future errors (Smith and Brewer, 1995; Siegert et al., 2014; Beatty et al., 2020). Such a process is thought to operate at least in part at the level of the decision threshold, increasing its height with respect to baseline activity as a means to augment the amount of (neural) evidence accumulation required to reach the decision threshold (Dutilh et al., 2012b; Purcell and Kiani, 2016; Schiffler et al., 2017; Fischer et al., 2018; Derosiere et al., 2018, 2019, 2022; Alamia et al., 2019); this, of course, prolongs the decision time but increases the probability of choosing the right circle and therefore the reward rate.

Consistent with the occurrence of such adaptive adjustment maximizing the reward rate in the hasty context, the PES observed there was associated with positive Δ%Correct values (Botvinick and Braver, 2015; Thura, 2020; Vassiliadis and Derosiere, 2020). Yet surprisingly, this was only true after misleading trial_n-1_ but not after ambiguous trial_n-1_, as Δ%Correct did not reach significance following the latter trial type. Hence, PES in the hasty context led to a gain in performance following misleading trial_n-1_ but not after ambiguous trial_n-1_. Such a finding suggests that errors did not solely trigger shifts in decision thresholds. Indeed, if this had been the case, one would have expected PES to be accompanied by a consistent increase in accuracy regardless of the type of trial_n-1_ in which an error occurred. Alternatively, a non-exclusive possibility is that task engagement varied following errors in these two trial_n-1_ types. We believe this may be the case because post-error task engagement (or arousal) has been shown to vary with the level of confidence at the moment an error is made (Yeung and Summerfield, 2012; Purcell and Kiani, 2016; Desender et al., 2019), which itself depends on the amount of sensory evidence available to make the (incorrect) choice (Meyniel et al., 2015; Pouget et al., 2016; Sanders et al., 2016; Urai et al., 2017; Desender et al., 2019). Accordingly, past studies have shown that when errors are made based on poor sensory evidence (i.e., with a low confidence level, as in ambiguous trials), arousal decreases significantly in the following trial (Notebaert et al., 2009; Nunez Castellar et al., 2010; Navarro-Cebrian et al., 2013; Purcell and Kiani, 2016; Wessel, 2018; Desender et al., 2019), possibly precluding an initially adaptive PES from leading to a significant gain in performance. By contrast, as previously observed incognitive interference tasks, when errors are related to the presence of high (conflicting) sensory evidence (as in misleading trials), arousal is found to increase in the following trial, an effect that may help dedicate attention to relevant sensory evidence (King et al., 2010; Danielmeier et al., 2011). Hence, it is plausible that in the current study, post-error task engagement was larger following misleading than ambiguous trial_n-1_, allowing PES to result in a performance gain following the former but not the latter trial type. Such a hypothesis could be tested in future work by investigating changes in pupil diameter following errors in our task (Kahneman and Beatty, 1966; Saderi et al., 2021).

Critically, Wessel proposed that PES arises from a sequence of processes including first a transient automatic response to the unexpected event (i.e., error), which triggers a reorientation of attention, followed by an adaptive process increasing the decision threshold to prevent future errors (Wessel, 2018). Based on this adaptive orienting theory, the delay between the feedback on trial_n-1_ (indicating an error) and the start of trial_n_, which corresponds to the intertrial interval(ITI) duration, can influence the nature of PES and needs to be long enough to allow the second adaptive process to take place. This was the case in our study where the ITI duration was 2,500 ms which, based on Wessel, is long enough for the adaptive process to occur. Hence, because the ITI duration was also comparable between the PES conditions, it is unlikely that this aspect of the task affected our data.

Unexpectedly, in this study, we observed PES only in the hasty but not in the cautious context. One tempting explanation is that slowing down after errors could only effectively increase accuracy in the hasty but not in the cautious context. That is because subjects emphasized speed in the hasty context, it is likely that a great proportion of errors were made because subjects responded too fast and not necessarily because the trial was difficult (Damaso et al., 2020). Hence, in this context, errors could easily be avoided by slowing down a bit in the following trial. In contrast, subjects were generally more cautious in the other context and it is thus plausible that errors occurred when choices were complex rather than because responses were too hasty (Ratcliff and Rouder, 1998; Brown and Heathcote, 2008; Ratcliff and McKoon, 2008). Slowing down following these trials may not be effective as it would not necessarily enhance accuracy; that is, even if subjects fail on the most complex choices, they are generally cautious enough to succeed on most trials and slowing down further would not lead to any performance gain. Yet, we believe such an explanation does not hold here. As such, it is important to note that RTs in cautious blocks were around 2,017 ms, which falls between Jump_10_ and Jump_11_, coinciding thus with the moment sensory evidence in favor of the correct choice starts to increase greatly (see Section “Material and Method”). This means that even if subjects were already generally cautious (and slower) in this context, slowing down would have been adaptive because it would have allowed providing responses based on more evidence.

A more plausible explanation is that the absence of PES in the cautious context is related to the way we promoted cautiousness in the current study. As such, changes in the SAT policy between the two contexts were engendered by manipulating the penalty size. However, even if error punishment is known to increase cautiousness (Potts, 2011; Derosiere et al., 2022), as desired here, monetary losses also generate an emotional response (Carver, 2006; Simoes-Franklin et al., 2010; Frijda et al., 2014; Eben et al., 2020b), a sense of frustration increasing with the size of the loss (Gehring and Willoughby, 2002; Holroyd et al., 2004; Yeung and Sanfey, 2004; Eben et al., 2020c). Importantly, such negative emotion has been shown to induce a post-error acceleration of RTs rather than a slowdown (Verbruggen et al., 2017; Dyson et al., 2018; Damaso et al., 2020; Eben et al., 2020c; Dyson, 2021). Accordingly, several studies have found that subjects act more impulsively after a loss or a non rewarded trial than a rewarded one (Gipson et al., 2012; Verbruggen et al., 2017; Eben et al., 2020c). Altogether, this literature suggests that the emotional response to monetary loss might have precluded us from observing PES in the cautious context. In other words, errors in the cautious context may have triggered opposite reactions counteracting each other; that is, a frustration feeling due to the high penalty (speeding up behavior) and an adaptive adjustment to prevent hasty errors (slowing down behavior). In the future, it would be interesting to dissociate the manipulation of the context from that of the penalty. Moreover, as the level of punishment sensitivity impacts error monitoring (Unger et al., 2012; Laurent et al., 2018), it also seems relevant to add some questionnaires to measure this personality trait such as the behavioral inhibition system (BIS) scale.

Interpretation of the current data is limited by the fact that a large number of subjects were excluded from the analyses because of an insufficient number of trials, reducing thus the sample size and the statistical power. In addition, the low error rate in the cautious context and the presence of different trial types impacted also the calculation of PES by preventing the use of another method than the traditional one. We recognize that this traditional method is prone to different biases such as global fluctuations in subject performance or in the number of post-correct trials outnumbering the number of post-error trials (Schroder et al., 2020). Note that even if some studies show that these biases can lead to an underestimation of post-error adjustments by decreasing effect sizes (Damaso et al., 2020; Schroder et al., 2020), others suggest that these biases do not radically change the results (van den Brink et al., 2014; Murphy et al., 2016).

In conclusion, our findings highlight a complex combination of processes that come into play following errors and that affect the speed of ensuing actions as well as the degree to which such post-error adjustment comes with a gain in performance or is rather maladaptive. The recruitment of these processes depends on several factors, including the context within which choices are made and the nature of erroneous trials, which affect altogether the subjects’ strategy, their engagement in the task, and likely also their emotional reaction to the error.

## Data Availability Statement

The raw data supporting the conclusions of this article will be made available by the authors, without undue reservation.

## Ethics Statement

The studies involving human participants were reviewed and approved by Comité d’éthique Hospitalo-Facultaire Saint-Luc—UCL. The patients/participants provided their written informed consent to participate in this study.

## Author Contributions

FF designed the study, analyzed the data, and wrote the first draft of the manuscript. GD acquired the data. FV and JD contributed to the study design and data analyses. All authors contributed to the article and approved the submitted version.

## Conflict of Interest

The authors declare that the research was conducted in the absence of any commercial or financial relationships that could be construed as a potential conflict of interest.

## Publisher’s Note

All claims expressed in this article are solely those of the authors and do not necessarily represent those of their affiliated organizations, or those of the publisher, the editors and the reviewers. Any product that may be evaluated in this article, or claim that may be made by its manufacturer, is not guaranteed or endorsed by the publisher.
